# Comparative genomics of the family *Vibrionaceae *reveals the wide distribution of genes encoding virulence-associated proteins

**DOI:** 10.1186/1471-2164-11-369

**Published:** 2010-06-10

**Authors:** Timothy G Lilburn, Jianying Gu, Hong Cai, Yufeng Wang

**Affiliations:** 1 Department of Bacteriology, ATCC, Manassas, VA 20110, USA; 2 Department of Biology, College of Staten Island, City University of New York, Staten Island, NY 10314, USA; 3 Department of Biology, University of Texas at San Antonio, San Antonio, TX 78249, USA; 4 South Texas Center for Emerging Infectious Diseases, University of Texas at San Antonio, San Antonio, TX 78249, USA

## Abstract

**Background:**

Species of the family *Vibrionaceae *are ubiquitous in marine environments. Several of these species are important pathogens of humans and marine species. Evidence indicates that genetic exchange plays an important role in the emergence of new pathogenic strains within this family. Data from the sequenced genomes of strains in this family could show how the genes encoded by all these strains, known as the pangenome, are distributed. Information about the core, accessory and panproteome of this family can show how, for example, genes encoding virulence-associated proteins are distributed and help us understand how virulence emerges.

**Results:**

We deduced the complete set of orthologs for eleven strains from this family. The core proteome consists of 1,882 orthologous groups, which is 28% of the 6,629 orthologous groups in this family. There were 4,411 accessory orthologous groups (i.e., proteins that occurred in from 2 to 10 proteomes) and 5,584 unique proteins (encoded once on only one of the eleven genomes). Proteins that have been associated with virulence in *V. cholerae *were widely distributed across the eleven genomes, but the majority was found only on the genomes of the two *V. cholerae *strains examined.

**Conclusions:**

The proteomes are reflective of the differing evolutionary trajectories followed by different strains to similar phenotypes. The composition of the proteomes supports the notion that genetic exchange among species of the *Vibrionaceae *is widespread and that this exchange aids these species in adapting to their environments.

## Background

Genomic comparisons among multiple strains of the same species have revealed that the overlap in gene content of any two strains is not complete, that is, the genomic resources of a species are not represented by the genome sequence of a single strain. In 2005 Tettelin et al. showed that the number of unique genes in seven genome sequences from *Streptococcus agalacticae*, which was termed the pangenome, far exceeded the number of genes found in any one strain and that this pangenomic repertoire would increase by at least 30 genes for every new genome of this species that was sequenced. Furthermore, it appeared that this increase would go on indefinitely [1]. The "infinite genome" phenomenon was not universal, for example, sequencing more than four genomes of *Bacillus anthracis *does not add any more genes to its pangenome. A terminology for classification of the genes found on sets of related genomes has been developed. As mentioned above the set of genes from all the genomes is called the pangenome (and the set of all encoded proteins is called the panproteome). The subset of these genes that is found on all the genomes is called the core genome and the set of genes that is found on more than one but not all genomes is called the accessory (or distributed) genome.

One of the original motivations for characterizing the pangenome was to understand what constitutes a bacterial species, but the value of such studies extends to understanding adaptation and evolution in the prokaryotes. Several studies have looked at pathogenic species and their non-pathogenic relatives in an effort to discover, for example, which genes might be unique to the pathogen and therefore drive pathogenesis (for example, see [2-6]. Few studies have looked at non-pathogenic relatives that lie outside the genus of the pathogen. Here we define a panproteome for the *Vibrionaceae*, a family containing several well-known human pathogens and species that play important roles in the marine ecosystem as nutrient cyclers, as partners in symbioses and as pathogens of fish and shellfish [7].

The most recent edition of Bergey's Manual of Systematic Bacteriology [8], divides the family *Vibrionaceae *into three genera encompassing 51 species. Since the appearance of that volume, several species of *Vibrio *have been moved into a new genus, *Aliivibrio*, and in 2006 Thompson and Swings estimated that the family included over 80 species [9]. This family provides a unique framework for examining the emergence of pathogenesis and the causes of virulence because of the combination of taxa it contains. Genome sequences from this family represent three species of human pathogen each with a different modality of infection and clinical manifestation: *Vibrio cholerae*, *V. vulnificus*and *V. parahaemolyticus*. Two of the genomes are from strains of *V. cholerae *that are both agents of pandemic cholera (strains O395 and N16961). The biotype represented by N16961, El Tor, is a so-called seventh pandemic strain. The El Tor biotype recently supplanted the sixth pandemic strains (represented by the classical biotype O395 strain) as the primary cause of pandemic cholera. Two more genomes represent *V. vulnificus *(strains CMCP6 and YJ016). This organism causes septicemia and the infections are rapidly fatal in persons having high levels of iron in their serum. A fifth genome represents *V. parahaemolyticus*, which causes gastroenteritis. Infection is usually associated with the ingestion of raw or undercooked seafood. Three more genomes represent strains pathogenic to marine organisms. *V. harveyi *has been identified as a pathogen of coral and shrimp [10], *V. splendidus *is a pathogen of oysters, mussels and scallops [11] and *Aliivibrio salmonicida *is the causative agent of Hitra disease in salmonid species [12]. Marine pathogens from the *Vibrionaceae *can have a serious impact on aquaculture operations [7]. The remaining three genomes are all non-pathogenic strains. Two genomes represent *A. fischeri *(strains ES114 and MJ11), a species that, like other *Vibrionaceae*, forms commensal relationships with fish and squid [13]. ES114 was isolated from squid, where it colonizes a specialized light organ, while MJ11 was isolated from a fish [14]. Finally, the eleventh genome represents a species that is not known to be either pathogenic or host-associated - *Photobacterium profundum *strain SS9. This species is notable for its ability to thrive at high pressure [15].

Although notable for the characteristics mentioned in the previous paragraph, many members of the *Vibrionaceae *can be isolated from more than one niche; often they are free-living as well as associated with one or more hosts. For example, *V. cholerae *is free-living, but can form biofilms on the exoskeletons of marine invertebrates such as shrimp, or colonize the trophozoites of amoeba [7,16], or, of course, it can survive and grow in the human intestine. Moving between these niches requires physiological adaptability and it is clear that this adaptability involves the sharing of genetic material among strains. For example, although *V. cholerae *is known as a disease-causing bacterium, only about 0.6% of the *V. cholerae *strains that can be detected in the environment are capable of producing the cholera toxin [17]. Similarly, a survey by Rahman et al. indicated that only a relatively small fraction of *V. cholerae *strains in the environment have all the genes needed for the pandemic strain phenotype, but relatively large numbers of strains have some of the genes that drive this phenotype [18]. For example, 79% of the environmental strains and all the clinical strains examined in their study carried the *hyl*A gene, which encodes hemolysin, an accessory virulence factor. One of the most common carbon sources in the marine environment, chitin, also induces the uptake of extracellular DNA by *V. cholerae*, adding support to the notion that genetic exchange is part of the adaptive strategy of the *Vibrionaceae *[19,20] and Udden et al. have demonstrated the transfer of the CTX phage genome from a non-toxigenic environmental strain to an O1 El Tor strain in the presence of chitin [21]. Gene flow within the species *V. cholerae *was recently examined by Chun et al. who found that the pangenome contains 2,432 orthologs shared by all 23 *V. cholerae *strains examined and 6,953 non-redundant genes in total [22]. They identified 12 lineages and propose that horizontal gene transfer largely drives diversification. A recent report indicating that a more virulent form of cholera is emerging and is caused by a recombinant O1 El Tor strain [23] underscores the need to explore the nature of the genetic pool available to the *Vibrionaceae *beyond a single species or genus. This knowledge will help us to better understand how and perhaps when, such new threats can arise. Here, we define and explore the panproteome of the family *Vibrionaceae*.

Within the context of the family-level panproteome, we examine how virulence-associated proteins from *V. cholerae *N16961 are partitioned among members of the family. We find that many of the proteins are widely distributed across the family, leading us to hypothesize that members of this family represent a resource for the genetic diversity of *V. cholerae *and the other pathogens. We also find genes that are unique to *V. cholerae *and examine their evolutionary history. Using these approaches we can better understand the evolutionary forces driving the emergence of pathogenic strains. On a practical level, in order to control or eliminate pathogenic strains, the genomic repertoire available to a species must be defined so that any measures devised towards these ends are effective against all the strains in a species [24].

## Results and Discussion

### The core, accessory, and panproteome of the Vibrionaceae

**Figure 1:**
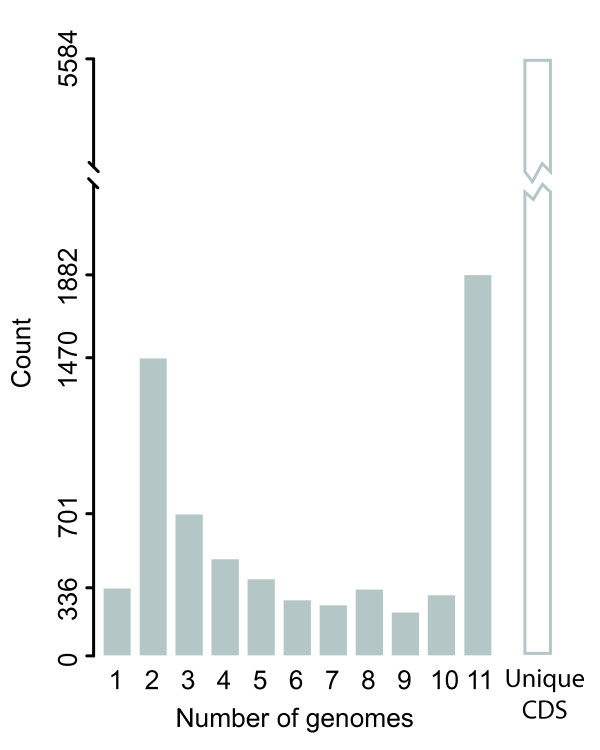
**Frequency distribution of orthologous groups in the eleven genomes examined**. The grey bars represent orthologous groups, while the white bar represents strain-unique proteins. 1,882 of the 6,629 orthologous groups are found in each of the 11 genomes. Only 336 of the orthologous groups are found in individual genomes. In addition to the orthologous groups, the pangenome contains 5,584 proteins that occur only once in one of the genomes.

Previously, we generated a complete set of orthologous proteins from the eleven strains of *Vibrionaceae *mentioned above [25]. In that study, we identified and evaluated instances of lineage-specific expansion within this family. Here, we evaluate this set of orthologs in the context of the pangenome of the family in terms of the distribution and function of the orthologs. The pangenome contained 49,588 protein coding sequences (CDS). 44,004 (88.7%) of the CDS fell into 6,629 orthologous groups (see Additional file 1 Table S1). A strain-by-strain breakdown of orthologous groups and CDS is given in Table 1. 1,882 of these orthologous groups were found in all 11 genomes studied and thus constitute the core proteome of the family *Vibrionaceae*. The accessory proteome consisted of 4,411 orthologous groups. The set of strain-specific proteins included 5,584 unique CDS plus 336 orthologous groups that each occurred in only a single genome. Overall, the panproteome consisted of 12,213 orthologous or unique sequences. There are few studies of the core and panproteomes of taxa at the genus or family levels. An analysis of 26 strains from the genus *Streptococcus *showed the core genome of this genus to be 611 orthologs [26]. Although this seems to be a small number it actually represents 26 to 36% of any one of the 26 genomes and therefore quite consistent with our results. A study of the core proteome of two families, the *Bacillales *and the *Enterobacteriaceae*, used a method for identifying core genome elements that incorporates genomic structure (gene order) as well as gene conservation [27]. The *Enterobacteriaceae *core proteome was estimated at 2,125 orthologous groups, or from 43 to 88% of any one of the 6 genomes analyzed. The *Enterobacteriaceae *are closely related to the *Vibrionaceae *but appear to have a more conserved set of proteins. The more distantly related *Bacillales *seem to have a more diverse set of proteins encoded in the pangenome, but the number of genomes included in this study may be too small to accurately estimate the true range of diversity in the two families. For the *Vibrionaceae*, the percentage of CDSs in each genome that was non-orthologous ranged from 5 to 21%. As each of these non-orthologous proteins could represent a different function, it is likely that these proteins represent a large part - 48.5% - of the functional diversity in the panproteome. This is illustrated in Figure 1, which shows the distribution of the orthologous groups among the 11 genomes. The largest group of orthologs is shared by all 11 genomes, but the potential functional diversity of the unique strain-specific CDS is equal to the functional diversity represented in the core proteome.

**Table 1:** Coding sequences, orthologous proteins, and their frequency in the 11 *Vibrionaceae *genomes used in this study

Strains	No. genes in genome	No. CDS	No. orthologous groups in strain	% CDS found in core genome	% CDS found in orthologous group	Orthologous groups unique to:	
						strain	species
*Vibrio cholerae *El Tor N16961	4009	3887	3433	49 (1899)	91 (3525)	11 (25)	319
*V. cholerae *O395	3998	3878	3537	49 (1902)	95 (3684)	49 (102)	319
*V. parahaemolyticus *RIMD 2210633	4708	4548	3977	42 (1918)	90 (4095)	24 (49)	24
*V. vulnificus *CMCP6	4796	4796	4122	40 (1910)	90 (4290)	16 (65)	318
*V. vulnificus *YJ016	4897	4758	4153	40 (1907)	90 (4284)	11 (24)	318
*V. harveyi *ATCC^® ^BAA-1116™	6238	6040	4116	32 (1920)	84 (5065)	97 (469)	97
*V. splendidus *LGP32	4604	4431	3718	43 (1921)	87 (3873)	13 (44)	13
*Aliivibrio fischeri *MJ11	4175	4039	3540	48 (1918)	90 (3642)	10 (21)	135
*A. fischeri *ES114	4038	3882	3451	50 (1921)	91 (3540)	3 (11)	135
*A. salmonicida *LFI1238	4352	3839	3143	50 (1919)	95 (3664)	12 (124)	12
*Photobacterium profundum *SS9	5702	5489	3762	35 (1932)	76 (4341)	90 (322)	90

The table presents the number of protein coding sequences (CDS) in each of the 11 *Vibrionaceae *genomes used in the comparative analysis, the numbers of orthologous groups found in each strain and the number of CDS encoding proteins in the core proteome. The numbers in brackets are the absolute counts. The inter-genomic search yielded a core genome comprised of 1,882 orthologous proteins.

### Functional classification of orthologous groups

**Figure 2:**
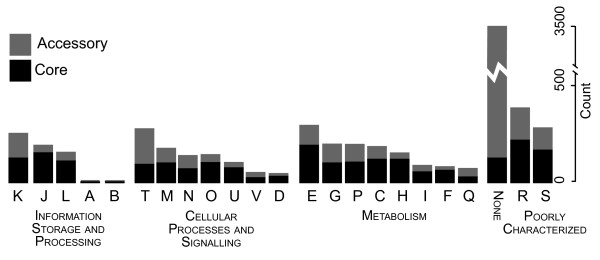
**Distribution of the 6,629 orthologous groups into COG functional categories**. The number of orthologous groups in each COG category is represented by the bar height. The proportion of each COG category that is composed of core (black) or accessory (grey) orthologous groups is shown. Categories are arranged within each class in order of size. In most categories, at least half the orthologs are found in the core proteome, but COG category T (Signal transduction mechanisms) is overrepresented in the accessory proteome.

The functions of the proteins in the 6,629 orthologous groups were estimated by classifying them into COG classes [28]. Figure 2 shows how orthologs in each class are divided between the core and accessory proteomes. Both core and accessory orthologs occasionally appear in more than one COG class. Over 93% of the core orthologs could be placed in COG classes. Some of these COG classes are disproportionately represented in the core proteome, including the so-called house-keeping proteins in COG classes J (Translation and associated functions), L (DNA replication, recombination and repair), U (Intracellular trafficking, secretion, and vesicular transport), and O (Posttranslational modification, protein turnover, chaperones), as well as proteins involved in central metabolism like classes E (Amino acid transport and metabolism), F (Nucleotide transport and metabolism), H (Coenzyme metabolism), and I (Lipid metabolism). The over-represented COG classes in the core proteome comprise the functionalities essential to the survival of the organisms.

Of the accessory orthologs, less than 24% could be classified successfully. This is to be expected since members of this set of proteins are taxon-specific and therefore more likely to be little known. Relative to the core proteome, the accessory proteome was enriched for only one COG class, T (Signal transduction mechanisms). Signal transduction enables each strain to sense and adapt to its environment. One class of such proteins, the methyl accepting chemotaxis proteins (MCPs), is known to be over-represented in the proteomes of the *Vibrionaceae*. The Microbial Signaling Database [29] contains information on 777 prokaryotes and the median number of MCPs per strain is three; if we consider only the 485 prokaryotes that have at least one MCP, the median number per strain is 12; in the *Vibrionaceae *the number of MCPs per genome ranges from 17 to 52.

We did not attempt to place the single copy CDS into COG groups, and only one of the 336 orthologous groups that were strain-specific could be placed in a COG class. When the annotations for proteins unique to *V. cholerae *N16961, which is the best studied of the strains in the *Vibrionaceae*, were retrieved from NCBI, only about 5% of the unique CDS had been placed in a COG class. The functionalities of the 5,584 strain-specific CDS from the other strains are likely to be equally obscure.

### Distribution of orthologous groups across the eleven genomes

**Figure 3:**
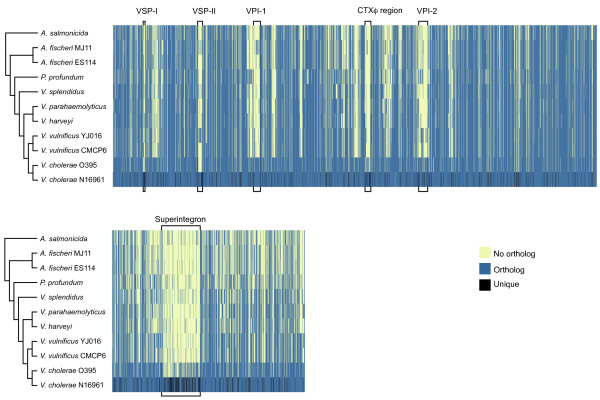
**Heatmap of core and accessory orthologs found in *V. cholerae *N16961 and their distribution among the sequenced *Vibrionaceae *strains**. The large (top) and small (bottom) chromosomes of the 11 strains are represented. Vertical blue or yellow bars represent the presence or absence, respectively, of each orthologous group in each genome. Vertical black bars represent proteins that are unique to *V. cholerae *N16961. The orthologous groups are arranged according to the encoding gene order on the *V. cholerae *N16961 chromosomes. Previously characterized genomic islands and the superintegron region are highlighted. The genomes are ordered top to bottom according to their phylogenetic relationships as shown in the dendrogram on the left. The dendrogram was calculated using the dollop algorithm from PHYLIP (see Methods) and is based on the presence or absence of the accessory orthologous groups.

Treating the presence or absence of an orthologous group from the accessory proteome in the genome of each strain as a binary character state and using these data as input to the dollop program from PHYLIP [30], we deduced the evolutionary relationships between the strains. The results are shown in Figure 3. The tree is consistent with evolutionary trees estimated using various conserved genes from the *Vibrionaceae *[31]. The distribution of the orthologous groups across the 11 proteomes, in the context of the *V. cholerae *proteome, is also shown in Figure 3. By arranging the strains according to their phylogeny, we can see the distribution of the orthologs in an evolutionary context.

It is obvious from Figure 3 that the distribution pattern of the orthologs does not always follow the path of vertical descent - clearly horizontal gene transfer has occurred. Several surveys of the genetic make-up of environmental and clinical strains of *V. cholerae *have shown that most of the genetic variability is found on genomic islands (see, for example, [22,32]). Figure 3 highlights the genomic islands, and the relative paucity of orthologs for proteins found encoded on these islands underscores their unusual nature; proteins encoded on the more "stable" regions of the large chromosome are more widely distributed. Genomic islands VSP-I and VSP-II are the *Vibrio *seventh pandemic regions and are found in the serotype O1 El Tor strains like N16961, but not on the sixth pandemic O1 strains like O395, although the insertion sites for the islands do exist on this strain. The absence of these elements is evidence that the proteins encoded on these elements are not required for the epidemic phenotype. Interestingly, all four of these genomic islands can excise from the genome and form circular intermediates [33,34]. Furthermore, the insertion site for these genomic islands can be found on other species of the *Vibrionaceae *[35]. The VSP-II insertion site, for example, brackets a much larger genomic island found in *V. vulnificus *YJ016 (where it is known as VVI-I, see Additional file 4 Figure S3) [36]. The VPI-1 insertion site, which brackets genes relevant to TCP expression, encloses completely different sets of genes in *V. vulnificus *strains YJ016 and CMCP6, in *V. parahaemolyticus *strain RIMD2210633, in *A. fischeri *E114 and in *P. profundum *SS9 [35].

While in the large chromosome the genomic islands appear to contain most of the genomic diversity, the small chromosome is more uniformly mixed, as reflected by the mosaic of colors. On this chromosome we observe that the pattern expected from vertical descent, an ortholog gradient from *V. cholerae *N16961 through to *A. salmonicida*, is more obvious, although the superintegron stands out as a region of intense diversification.

### Virulence-associated orthologous groups

**Figure 4:**
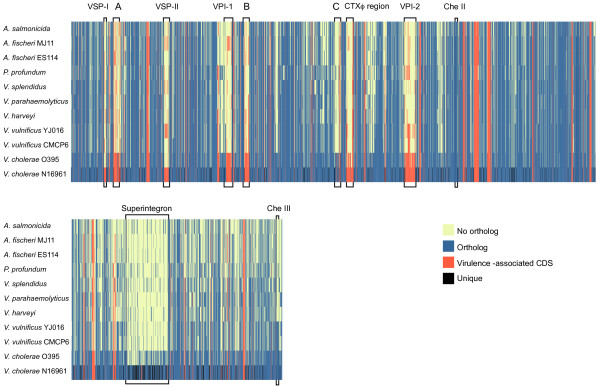
**Distribution of virulence-associated orthologous groups across eleven *Vibrionaceae *genomes**. The presentation is as in Figure 3, except the dendrogram is eliminated to save space and virulence-associated proteins (both unique and orthologous) are shown as vertical red bars. In addition to the well-known genomic islands, three regions (A, B, and C) that were identified in this study and are discussed in the text are shown, along with the Che Cluster II and III regions. These regions are marked Che II and Che III respectively. Che Cluster I is within region C.

We have published lists of virulence-associated genes and candidate virulence associated genes from *V. cholerae *N16961 [37]. We identified 526 virulence-associated proteins in this strain from the literature and databases. A further 463 proteins were identified as candidate virulence-associated proteins via a protein-protein association network analysis. As some of these proteins were found in orthologous groups, the total number of unique proteins and orthologs was 913; the distribution of this set of virulence-associated proteins across the 11 *Vibrionaceae *genomes is visualized as a heat map in Figure 4. As in Figure 3, arranging the orthologs according to the sequence of one of the strains eliminates visualization problems caused by genome rearrangements (synteny). It is easy to score the presence or absence of all the virulence-associated orthologs from *V. cholerae *N16961 in this way. One hundred and eighty-six of the virulence-associated proteins are in the core proteome of the *Vibrionaceae *and 295 are in the accessory proteome. Only 22 proteins are unique to *V. cholerae *N16961. Among the candidate virulence-associated proteins, 247 are in the core proteome, 185 are in the accessory proteome and 23 are unique to *V. cholerae *N16961.

Aside from the recognized genomic islands marked in Figure 4, we can recognize at least three other regions on the large chromosome, marked A, B, and C, that encode virulence proteins that are unique to *V. cholerae*. Regions A and B are associated with cell-surface polysaccharides. Region A (VC0245-VC0250) encodes gene products required for the synthesis of the O-type antigens, which give the two strains of *V. cholerae *their O1 serotype. Loss of the ability to synthesize an O-antigen, for example, by disruption of VC0249 (*rfb*L), has been shown to severely impair *V. cholerae*'s ability to establish an infection in mice [38]. A search for homologs of the six region A proteins in the UniRef50 database [39], which clusters proteins on the basis of a sequence identity of 50% or greater, revealed that for all six proteins the other members of their respective clusters were all from other serotype O1 strains of *V. cholerae *and had a sequence identity of < 99%. A more extensive search in the IMG database [40] showed that the best match homologs with less than 50% identity were from outside the family *Vibrionaceae*.

Region B (VC0926-VC0933) includes proteins involved in biofilm formation. Loci VC0926 and VC0927 are part of the *vps*I cluster (Vibrio polysaccharide synthesis) [41]. Loci VC0928 through VC0933 (also known as *rmb*A through F [42]) are interposed between *vps*I and a second *vps *cluster, *vps*II. Biofilm formation is a regulated response to environmental conditions and the *rmb *genes are all expressed when the *vps*I and *vps*II clusters are up-regulated [43]. Deletion of any of them suppresses the formation of the biofilms associated with *vps *gene expression [42]. Expression of the *Vibrio *polysaccharide (VPS) genes has been linked to increased survival of toxigenic strains challenged with chlorine (and is thus an important trait for survival in municipal water supplies) [44], changes in osmotic pressure and pH and oxidative stress [41,45,46]. Furthermore, *in vivo *experiments have linked the biofilm formation associated with the VPS protein expression to virulence [47,48]. All of the most similar orthologs to VC0926 and VC0927 are found in various serotypes of *V. cholerae*; outside this species, the genus *Burkholderia *has homologs with over 60% identity, but there are no orthologs in any other *Vibrionaceae*. Orthologs of VC0928 appear to be restricted to *V. cholerae*. Orthologs of VC0930, a putative hemolysin do not occur in any other *Vibrionaceae *species, although some strains of *V. cholerae *have more than one ortholog. VC0931, VC0932 and VC0933 are confined to *Vibrio *species, but low-identity orthologs of VC0931 and VC0932 are seen in many genera. Like the O-antigen, the proteins encoded by *vps*I and by the *rbm *genes appears to be unique to *V. cholerae*, but not only in strains with the virulent, toxigenic phenotype. Thus, these proteins count among the accessory virulence proteins and play a dual role, as they also help *V. cholerae *survive outside the host. Interestingly, the genes encoded on the *vps*II cluster are found in other members of the *Vibrionaceae*, including *A. fischeri*, *V. harveyi*, *V. parahaemolyticus*, and *V. vulnificus*. In the latter species, three of the *vsp*II genes (orthologs of VC0934, VC0936, and VC0937) are duplicated, a lineage specific expansion that we speculate was mediated via horizontal gene transfer [25].

Region C (VC1394-VC1406) encloses one of three clusters of genes on the *V. cholerae *chromosomes that are paradigmatically involved in chemotaxis. The clusters are designated clusters I through III [49] and region C includes the cluster I chemotaxis genes. Clusters II and III are found on chromosomes 1 and 2 respectively (Figure 4). Only the proteins encoded in cluster II (VC2059-VC2065) can be shown to be involved in chemotactic signaling *in vitro *[50,51]. Although the histidine kinase (CheA-1) and response regulators (CheY-1 and CheY-2) encoded in region C are homologous to the cognate proteins in cluster II, they cannot complement cluster II deletion mutants. CheY-1 and CheY-2 are each missing amino acid residues involved in the interaction between CheY and FliM [51,52]. FliM is part of the flagellar assembly and mediates a change in rotational direction of the flagellum, which is important to chemotaxis. Phylogenetic analysis of the Che cluster I proteins shows that, within the *Vibrionaceae*, they are exclusively found in *V. cholerae *strains (Additional file 2 Figure S1). In addition, with the exception of CheA, they are more similar to the Che proteins encoded in cluster III than to those encoded on cluster II. Gene neighborhood analysis using the IMG resource [40] shows that the cluster I homologs always group together on the large chromosome. Interestingly, the four MCP homologs flanking the Che homologs in region C are, like the CheY-1 and -2 homologs, unusual. While 38 of the 45 the MCPs found in *V. cholerae *are members of the 40H group, as classified by Alexander and Zhulin [29,53], only one of the four MCPs in region C is a member of this class. The two MCPs (the VC1394 and VC1403 orthologs) immediately flanking region C each have unusual domain architectures. VC1394 orthologs are classified as Unaligned Membrane-bound (UM) MCPs, indicating that they are membrane bound but lack an identifiable sensory domain. UM MCPs are found in many strains, but their function is not known. VC1403 homologs are from group 44H and have two MCP signal domains; there is only one 44H MCP in each *V. cholerae *genome. The expression of the MCP encoded by VC1403 appears to be enhanced under anaerobic conditions [54] similar to those found in the intestine. The last protein in region C, VC1406, is a 24H MCP. This group has no obvious methyl binding sites, sites that are important for signal transduction. One possibility is that this type of MCP modulates signal amplification within MCP arrays. The unusual nature of the MCPs and Che proteins found in region C hints at functions other than chemosensing-driven motility. Their potential involvement in virulence is indicated by at least two studies. In the first, transcription of genes in this region was shown to be increased under conditions designed to induce expression of virulence genes [55] and in the second VC1397 and VC1399 were shown to be expressed in the course of human infections [56].

Region C is part of a 168 kb cluster identified using M-GCAT [57] (Additional file 3 Figure S2). Alignment of this cluster using the MUSCLE algorithm [58] bundled with M-GCAT showed that there was 99.5% identity among the sequences from all the complete *V. cholerae *genomes currently available. Region C is also conserved in the draft *V. cholerae *genomes available through IMG. Recently, Chun et al. identified the region spanning loci VC1393 to VC1405 as a genomic island [22] and it largely overlaps region C of Figure 4. Their analysis of the distribution of this region is consistent with ours. Like other putative genomic islands, it does not partition among strains of *V. cholerae *strictly according to pathogenicity. Nonetheless, it is invariably present in seventh pandemic strains and is in fact only absent from a single strain that has a CTXphi region, that is, V51, a clinical isolate from the United States. This supports the idea that the region C orthologs have a role in epidemic virulence.

### Functions of virulence-associated orthologs

The two *V. cholerae *strains share 319 orthologous groups that are not found in the other strains. Only 62 of these orthologs are classified as virulence proteins and most of them are found in the regions marked in Figure 4. The fact that so few putative virulence genes are unique to these two strains testifies to the wide distribution of virulence-associated genes among the *Vibrionaceae*. Just over one third of the shared virulence-associated orthologs were placed in a COG functional group. COG classes T and N are over-represented in this set. Of course, the orthologs encoded in region C are members of these COG classes and exemplify some of the signaling and regulatory processes that are doubtless required for successful colonization of the human host. The high proportion of unclassified orthologous groups in the set of virulence-associated orthologs unique to *V. cholerae *represents a pool of unrecognized functionalities that may equip the species to survive and flourish in the environments it faces. For example, there are four hypothetical proteins, loci VCA0881 through VCA0884 in strain N16961, that appear to form an operon [59]. When a search of the National Microbial Pathogens Data Resource (NMPDR) was done, two of these proteins were found to be annotated as non-hemolytic enterotoxin lytic components. These proteins are found in other strains of *V. cholerae*, but not in any other species of *Vibrionaceae*. The most similar proteins outside this species are the non-hemolytic enterotoxins encoded by some *Bacillus *species, and are known to contribute to the pathogenic effects of these species.

It is surprising that these two strains of *V. cholerae *do not share more virulence proteins. One possible explanation is that their virulence, while employing the same CTXphi and toxin co-regulated pilus genes, are built on different underlying adaptations to human host-associated selective pressures. Both are O1 serotype strains, but O395 is a Classical biotype strain (also known as a sixth pandemic strain) while N16961 is an El Tor biotype strain (also known as a seventh pandemic strain). The two strains appear to have arisen independently. A recent analysis by Feng et al. [60] suggests that the mutation rate in *Vibrio *species is much higher than originally thought and that the El Tor biotype acquired its capability to become a pandemic strain independently of O395. The adaptations that arose in N16961 on its evolutionary path to virulence have allowed it to displace O395 as the causative agent of cholera around the world. A similar situation has been inferred in *Escherichia coli*, pathogenic strains of which are thought to have arisen more than once [3]. Thus the complete set of genes involved in the cholera virulence phenotype may be quite different in the two strains, with only a subset of the potential virulence factors used in each strain.

Looking more widely at the *Vibrionaceae *we find there are 943 orthologous groups that contain only proteins from human pathogens (*V. cholerae*, *V. vulnificus *and *V. parahaemolyticus*) and 1,907 orthologous groups that contain only proteins from pathogenic strains (the human pathogens plus *V. harveyi*, *V. splendidus *and *A. salmonicida*). It would be misleading to assess these shared sets of genes in terms of virulence potential as these species have different modalities of infection and virulence. However, rearranging our core and accessory set of orthologs in the context of, for example, the genome of *V. vulnificus *YJ016 immediately reveals sets of orthologs that are unique to the species.

Twelve genomic islands have been defined for *V. vulnificus *YJ016 and they are shown in Additional file 4 Figure S3 [35,36,61]. Orthologs to the proteins encoded on these genomic islands exist on other strains (See Additional file 4 Figure S3), but, as discussed above, frequently the insertion site containing a genomic island in *V. vulnificus *YJ016 will enclose a different set of genes in another species. Regions VVI-I through VVI-IX meet the canonical requirements for genomic islands as defined in the literature [62] and six of them were found to be unique to strain YJ016 [35]. The regions VVI-X through VVI-XII are found in strains YJ016 and CMCP6, but not in other species. These regions do not have all the features normally associated with genomic islands, but they do seem to show a presence/absence pattern across strains of *V. vulnificus *that indicates they are mobile. The presence of region VVI-XII was found to correlate with strains of *V. vulnificus *that were, or had the genetic potential to be, clinical isolates [61]. Scanning Additional file 4 Figure S3, we can see other regions containing strings of orthologs unique to *V. vulnificus*. The orthologs encoded on loci VV1999 to VV2015 (YJ016 loci numbering) and marked as region A in Additional file 4 Figure S3 are a good example. The orthologs in this region span 15.6 kb. They include VV2003 to VV2012. Experiments examining the effect of changes in environmental concentrations of iron on transcription showed that these loci were induced under iron-limiting conditions and that they form an operon [63,64]. Miyamoto et al. also showed that the Fur (ferric uptake regulator) protein regulates the expression of these genes. Iron availability is known to play a major role in the virulence of *V. vulnificus *[65], but Alice et al. were not able to link these genes to virulence. Other experiments designed to elucidate the role of the AphB regulatory protein in *V. vulnificus *showed that AphB regulates the expression of the genes in region A, along with many others. AphB mutants are less virulent than wild-type strains [66], and show a reduced ability to adhere to host cells. Interestingly, VV2003 to VV 2012 encode protein domains associated with the formation of pili thought to be involved in adherence. With the exception of VV2007 (Flp pilus assembly protein TadA), the encoded proteins do not have over 30% identity with other proteins containing similar domains and may carry out novel adherence functions associated with iron-poor environments such as those usually found in human serum. We feel these orthologs merit further investigation, as they are unique to *V. vulnificus*, and may contribute to functions that make this species one of the deadliest food-borne pathogens known.

## Conclusions

The panproteome of the *Vibrionaceae *consists of 12,213 unique groups. 1,882 (15%) of these groups form the core proteome and 4,411 groups (36%) form the accessory proteome. These numbers are consistent with those reported for analyses of related groups at the species [22,67] and family levels [27] and are similar to a genus-level study from a more distantly related taxon [26]. This rather high level of sequence conservation is reflected in the conservation of so many virulence-related proteins from *V. cholerae *across the *Vibrionaceae*. It has been observed that the size (and potential size) of the panproteome, which is directly related to sequence diversity, appears to correlate with the life-style of the bacterium rather than with its taxonomic classification [68]. In the species *V. cholerae*, Keymer et al. [67] have shown that the non-homogenous marine environment drives the formation of diverse populations of *V. cholerae *strains, each influenced by the environment in which it lives. Similarly, *V. splendidus *has been shown to form distinct populations within different ecological niches in the marine environment [69]. Hence, although apparent niche-driven generation of diversity may oppose it, many of the genes associated with the virulence of *V. cholerae *are widely distributed within the *Vibrionaceae*. This conservation may not be a response to the need to survive in the human intestine, which many strain from this family never see, but a sign that the virulence-associated proteins are fitness factors required by *V. cholerae *and other *Vibrionaceae *to ensure their physiological flexibility. The marine environment allows niche specialization and the coexistence of a variety of genotypes, but it is also dynamic, and the barriers to genetic exchange among the genotypes are occasionally removed. *V. cholerae *can exchange genetic material via phage-mediated mechanisms, conjugation and chitin-induced natural competence [19]. The latter mechanism does not require special sequence elements or dedicated enzymology to achieve integration of donor DNA, and much of the necessary machinery is conserved not only with in the species *V. cholerae*, but across the *Vibrionaceae *(see Additional file 1 Table S1). We observed above that some genomic islands appear to move across the species boundary and we believe that movement across the genus boundary also occurs. High rates of genetic exchange can help species survive environmental challenges, as has been observed for *Streptococcus pneumoniae *[70] and this principle may operate for the *Vibrionaceae*, but presumably the high rates of recombination, which, in principle, should lead to genomic homogenization, are balanced by forces that ensure the species within the *Vibrionaceae *remain distinct [71]. Interestingly, Stine et al. recently showed that concurrent outbreaks of cholera in Bangladesh were caused by different genotypes of epidemic *V. cholerae *[72]. This supports the idea that the gene pool sustained by the *Vibrionaceae *aids in the emergence of multiple epidemic strains when conditions are favorable. Other species within the *Vibrionaceae *have their own unique sets of proteins, which can contribute to the pathogenic phenotype. Our approach facilitates the identification of such sets, highlighting proteins that, like the signal transduction systems discussed above, may play key roles in virulence.

## Methods

### Genomes and annotation

Eleven genomes from the family *Vibrionaceae *including *V. cholerae *strains O1 biovar El Tor str. N16961 [GenBank: NC_002505, NC_002506] [73] and O1 biovar classical str. O395 [GenBank: NC_009456, NC_009457] [22], *V. parahaemolyticus *RIMD 2210633 [GenBank: NC_004603, NC_004605] [74], *V. vulnificus *CMCP6 [GenBank: NC_004459, NC_004460] [75], *V. vulnificus *YJ016 [GenBank: NC_005139, NC_005140, NC_005128] [76], *V. harveyi *ATCC^® ^BAA-1116™ [GenBank: NC_009777, NC_009783, NC_009784], *V. splendidus *LGP32 [GenBank: NC_011744, NC_011753] [77], *Aliivibrio (Vibrio) fischeri *ES114 [GenBank: NC_006840, NC_006841, NC_006842] [78], *A. fischeri *MJ11 [GenBank: NC_0111866, NC_0111865, NC_0111864] [79], *A. salmonicida *LFI1238 [GenBank: NC_011312, NC_011313, NC_011314, NC_011311, NC_011315, NC_011316] [12], and *Photobacterium profundum *SS9 [GenBank: NC_006370, NC_006371, NC_005871] [15] were downloaded from the J. Craig Venter Institute's Comprehensive Microbial Resource [80].

Additional annotation for individual strains was retrieved from the National Microbial Pathogen Data Resource (NMPDR) [81] The UCSC Archaeal Genome Browser [82] the Integrated Microbial Genomes system [40] and from UniProt [83].

### Identification of orthologous groups

The procedure is described in Gu et al. [25]. Open reading frames from the genome sequences were analyzed using OrthoMCL [84] to detect and group the orthologous proteins in the 11 strains of *Vibrionaceae*. OrthoMCL is a good choice for ortholog detection as it has reasonably low false positive and false negative detection rates and can detect orthologs across a group of genomes [85]. From the results we identified three sets of proteins: (i) those that were encoded by all 11 strains, and which we call the core proteome of the *Vibrionaceae*, (ii) those encoded on two or more, but less than eleven of, the genomes. We refer to these as the accessory proteome, and (iii) those that were encoded on only one genome, which we refer to as the strain-unique proteome. Together these three sets compose the panproteome of the *Vibrionaceae*. A hierarchical functional classification of the proteins that fell into OrthoMCL groups was performed by searching against the Clusters of Orthologous Groups (COG) database [86].

### Phylogenetic analysis

We scored the presence or absence of all orthologs in each of the eleven genomes and used the accessory proteome to calculate the phylogenetic relationships among the 11 strains using the program dollop from the PHYLIP package [30]. Huson and Steel have demonstrated the superiority of Dollo parsimony over distance-based methods for the deduction of phylogenetic trees based on gene content [87]. The tree was drawn using Dendroscope [88]. The presence and absence of each ortholog in each genome was visualized using the R statistical package and the gplots library. Orthologs were ordered in the plots according to their occurrence in the genome of interest.

Phylogenetic analysis of the CheA, CheB, CheY and CheZ orthologs of the *V. cholerae *proteins was done as follows. First, the sequences of the orthologs were collected in fasta format from the UniProt database. Alignments were carried out using the L-INS-I method of the MAFFT software, version 6.713 [89] and evaluated using pfaat [90]. Maximum likelihood trees were inferred using the Treefinder software [91]. The Whelan and Goldman substitution model [92] was used.

## Authors' contributions

TGL, JG, and YW conceived and designed the study. All authors performed data analysis. HC wrote the scripts. TGL drafted the manuscript, YW, JG, and HC edited it. All authors read and approved the final manuscript.

## Supplementary Material

Additional file 1 **Table S1**. The orthologous groups found in 11 genomes from the *Vibrionaceae *The table shows a unique identifier for each orthologous group (ORTHO ID), the number of genomes in which it occurs (#Strains), the strains in which the orthologs occur (Strain Distribution), the number of copies of each orthologous protein found (#Seqs), functional information (COG Functional Description), the COG category (COG Cat), the COG family ID (COG Fam), and the identifiers for each occurrence of the orthologs (Identifiers). Locus identifiers that begin with NTO and VC are the JCV-CMR locus identifiers; the remaining identifiers are GenProt accession numbers. Click here for file

Additional file 4 **Figure S3**. Heat map of core and accessory orthologs found in *V. vulnificus *YJ016 and their distribution among the sequenced *Vibrionaceae *strains. Vertical blue or yellow bars represent the presence or absence, respectively, of each orthologous group in each genome. Vertical black bars represent proteins that are unique to *V. vulnificus *YJ016. The orthologous groups are arranged according to the encoding gene order on the *V. vulnificus *YJ016 chromosomes. The genomes are arranged as in Figure 3, according to their phylogenetic relationships as calculated from their shared orthologous groups content. The twelve recognized genomic islands in strain YJ016 are labeled along with the superintegron region. Region A, encompassing orthologs unique to the species *V. vulnificus*, is discussed in the text. Click here for file

Additional file 2 **Figure S1**. Phylogenetic relationships among the Che proteins of the *Vibrionaceae *Four maximum likelihood trees showing the phylogenetic relationships among the Che A, B, W, and Y homologs of the *Vibrionaceae *are shown. The homologs are named according to the nomenclature in [49], except that here the putative gene products are designated "-0". The clusters labelled in red are orthologs of the Che Cluster I protein. In all cases, these orthologs are found only in other *V. cholerae *strains. Clusters labelled in blue are orthologs of the Che Cluster II proteins. Che Cluster II orthologs are essential for chemotactic motility in *V. cholerae*. Outgroup sequences were selected from the *Gammaproteobacteria*. Sequences were aligned using mafft 6.713. Maximum likelihood trees were calculated using Treefinder 2008. Alignments and trees were visualized using pfaat 2.0. Click here for file

Additional file 3 **Figure S2**. Showing the location of the homologous cluster containing region C in the genomes of four strains of *V. cholerae*. The large chromosomes of four strains of *V. cholerae *were aligned using the G-MCAT program. The genomic section that includes region C is shown in each chromosome in light green. White bars in each chromosome represent the absence of genes that are not found in all the chromosomes. Click here for file
