# Supplementary figures and images for: Comparative genomics of the family Vibrionaceae reveals the wide distribution of genes encoding virulence-associated proteins
Source: BMC Genomics. 2010 Jun 10;11:369. doi: 10.1186/1471-2164-11-369 (PMC2890568; doi:10.1186/1471-2164-11-369)

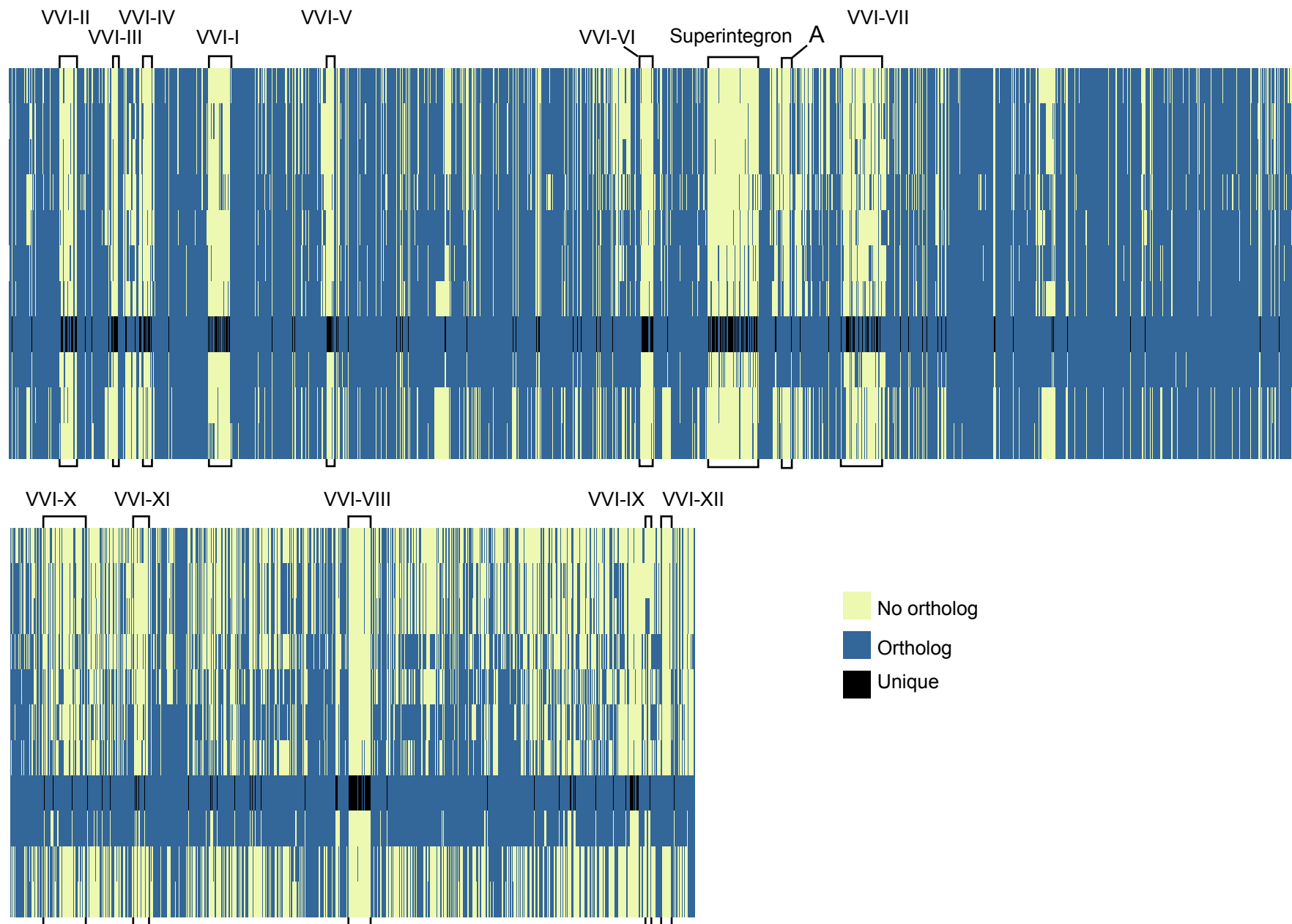

Supplement: Additional file 4 — Figure S3. Heat map of core and accessory orthologs found in V. vulnificus YJ016 and their distribution among the sequenced Vibrionaceae strains. Vertical blue or yellow bars represent the presence or absence, respectively, of each orthologous group in each genome. Vertical black bars represent proteins that are unique to V. vulnificus YJ016. The orthologous groups are arranged according to the encoding gene order on the V. vulnificus YJ016 chromosomes. The genomes are arranged as in Figure 3, according to their phylogenetic relationships as calculated from their shared orthologous groups content. The twelve recognized genomic islands in strain YJ016 are labeled along with the superintegron region. Region A, encompassing orthologs unique to the species V. vulnificus, is discussed in the text. [file 1471-2164-11-369-S4.PDF]

CheA orthologs

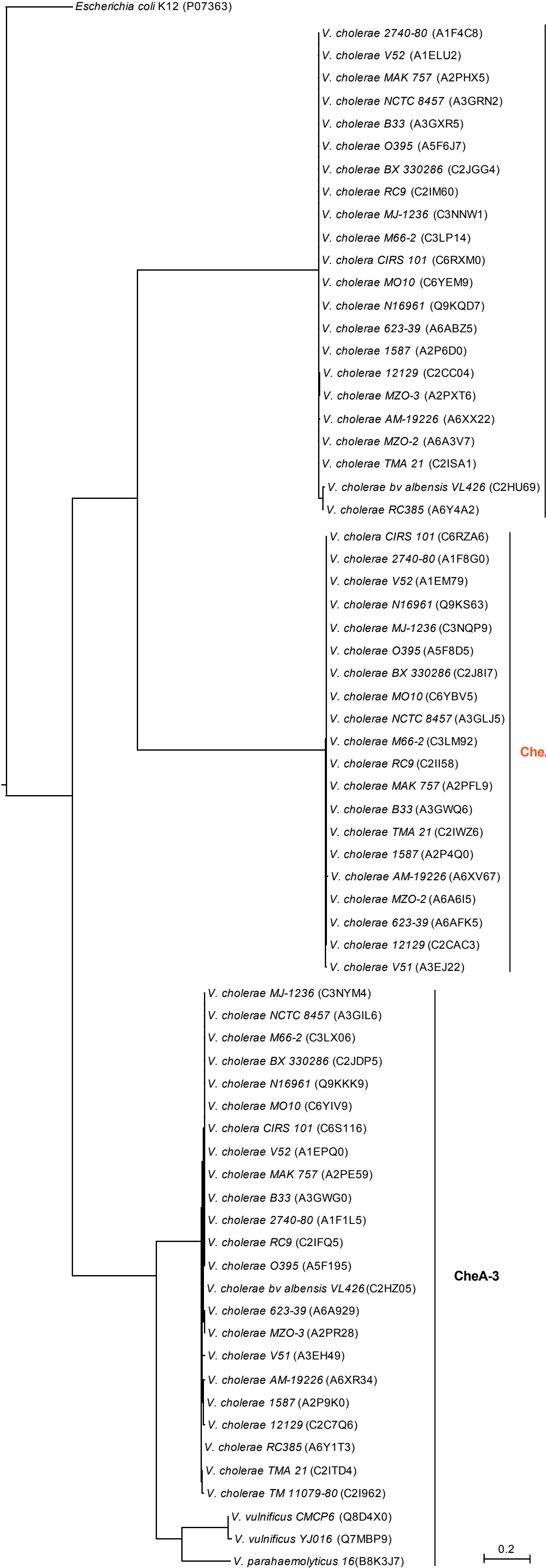

CheB orthologs

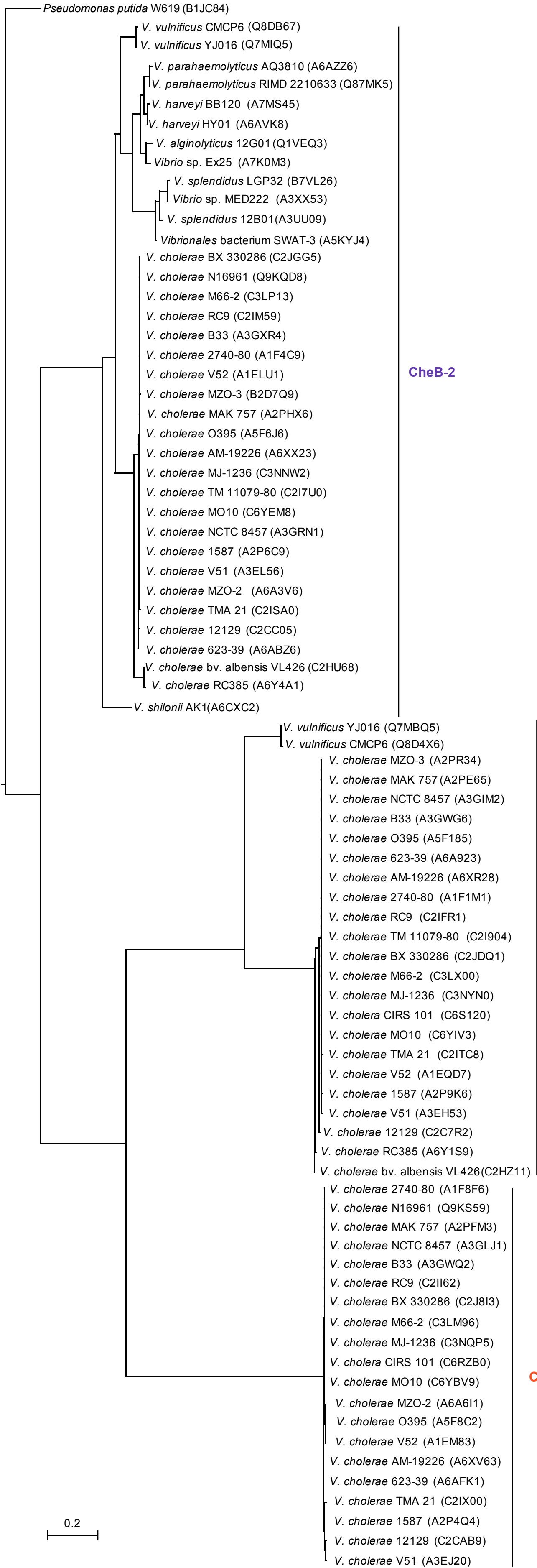

CheW orthologs

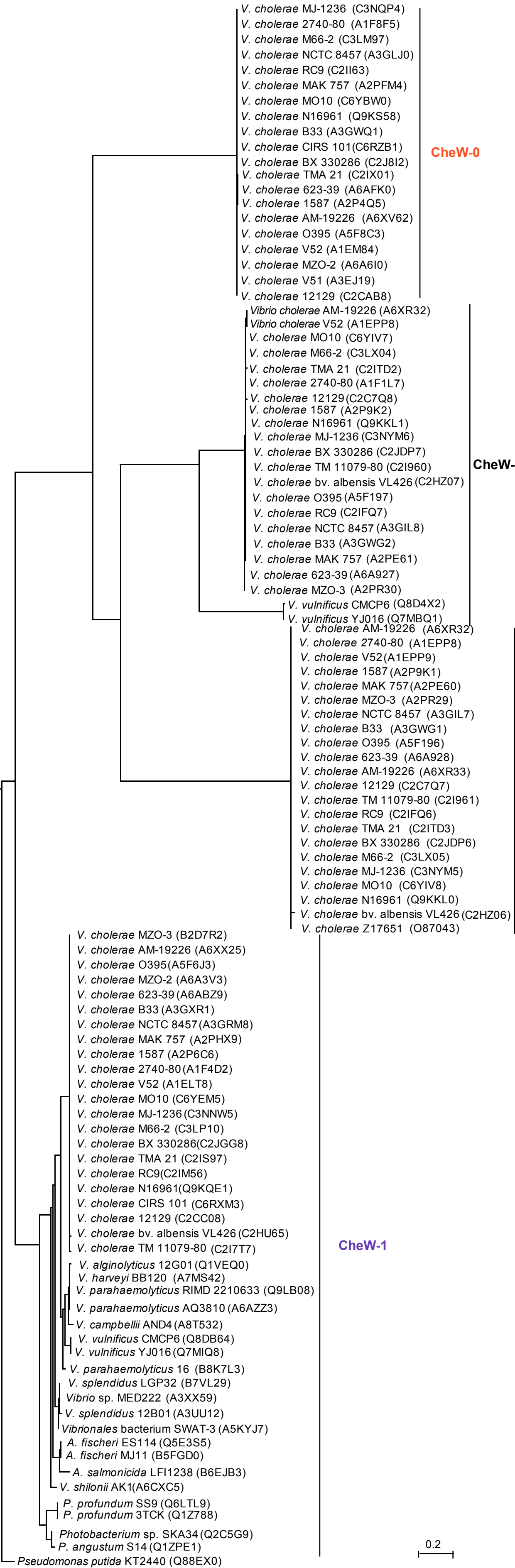

CheY orthologs

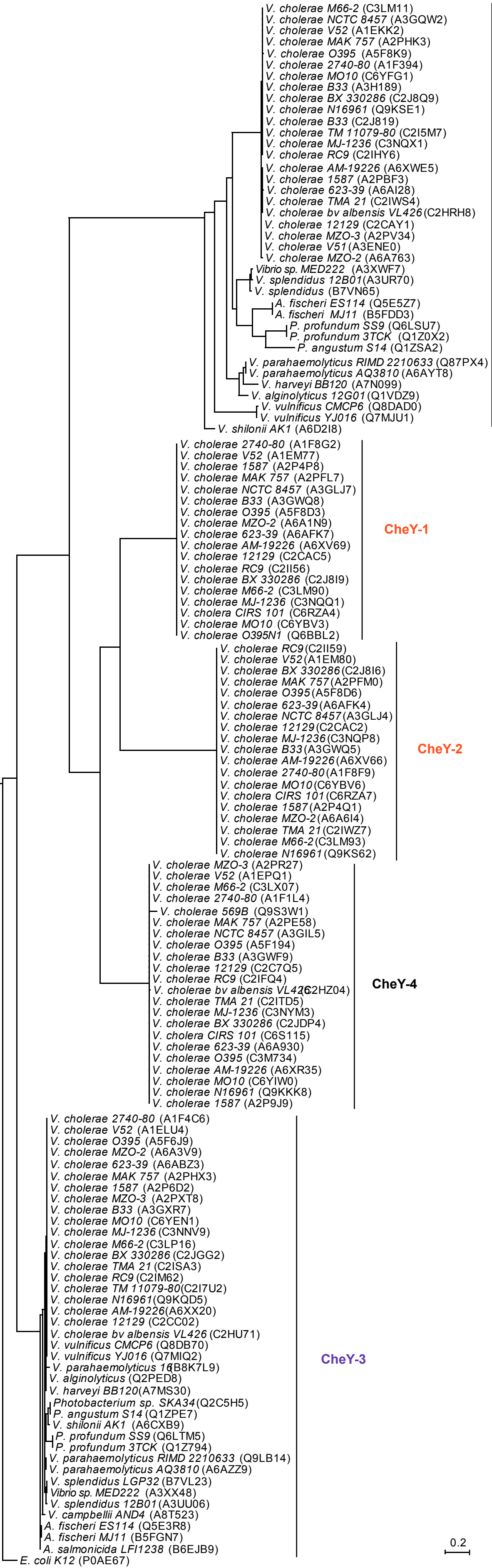

Supplement: Additional file 2 — Figure S1. Phylogenetic relationships among the Che proteins of the Vibrionaceae Four maximum likelihood trees showing the phylogenetic relationships among the Che A, B, W, and Y homologs of the Vibrionaceae are shown. The homologs are named according to the nomenclature in [49], except that here the putative gene products are designated "-0". The clusters labelled in red are orthologs of the Che Cluster I protein. In all cases, these orthologs are found only in other V. cholerae strains. Clusters labelled in blue are orthologs of the Che Cluster II proteins. Che Cluster II orthologs are essential for chemotactic motility in V. cholerae. Outgroup sequences were selected from the Gammaproteobacteria. Sequences were aligned using mafft 6.713. Maximum likelihood trees were calculated using Treefinder 2008. Alignments and trees were visualized using pfaat 2.0. [file 1471-2164-11-369-S2.PDF]

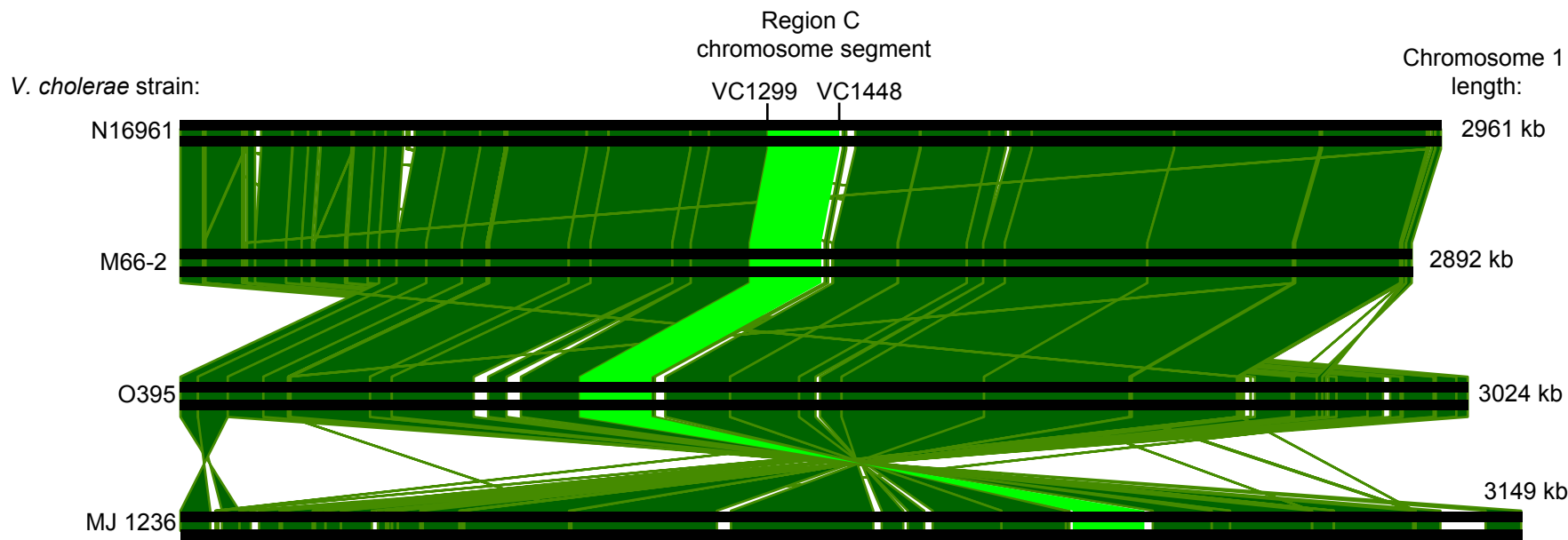

Supplement: Additional file 3 — Figure S2. Showing the location of the homologous cluster containing region C in the genomes of four strains of V. cholerae. The large chromosomes of four strains of V. cholerae were aligned using the G-MCAT program. The genomic section that includes region C is shown in each chromosome in light green. White bars in each chromosome represent the absence of genes that are not found in all the chromosomes. [file 1471-2164-11-369-S3.PDF]
